# PprI: The Key Protein in Response to DNA Damage in *Deinococcus*

**DOI:** 10.3389/fcell.2020.609714

**Published:** 2021-01-18

**Authors:** Huizhi Lu, Yuejin Hua

**Affiliations:** MOE Key Laboratory of Biosystems Homeostasis and Protection, Institute of Biophysics, College of Life Sciences, Zhejiang University, Hangzhou, China

**Keywords:** PprI, DNA damage response, switch, PprI-DdrO system, *Deinococcus*

## Abstract

Deoxyribonucleic acid (DNA) damage response (DDR) pathways are essential for maintaining the integrity of the genome when destabilized by various damaging events, such as ionizing radiation, ultraviolet light, chemical or oxidative stress, and DNA replication errors. The PprI–DdrO system is a newly identified pathway responsible for the DNA damage response in *Deinococcus*, in which PprI (also called IrrE) acts as a crucial component mediating the extreme resistance of these bacteria. This review describes studies about PprI sequence conservation, regulatory function, structural characteristics, biochemical activity, and hypothetical activation mechanisms as well as potential applications.

## Introduction

Deoxyribonucleic acid (DNA) damage occurs when the genome is exposed to exogenous and endogenous hazards, leading to imperfection and instability of the genetic information (Pilzecker et al., 2019). If not repaired in a timely and accurate manner, accumulating mutations will result in severe effects, such as cancer, and even lead to cell death. To cope with DNA damage, organisms have evolved various DNA damage repair pathways, including nucleotide excision repair, non-homologous end joining, homologous recombination, mismatch repair, and base excision repair (Boulton et al., 2002).

**FIGURE 1:**
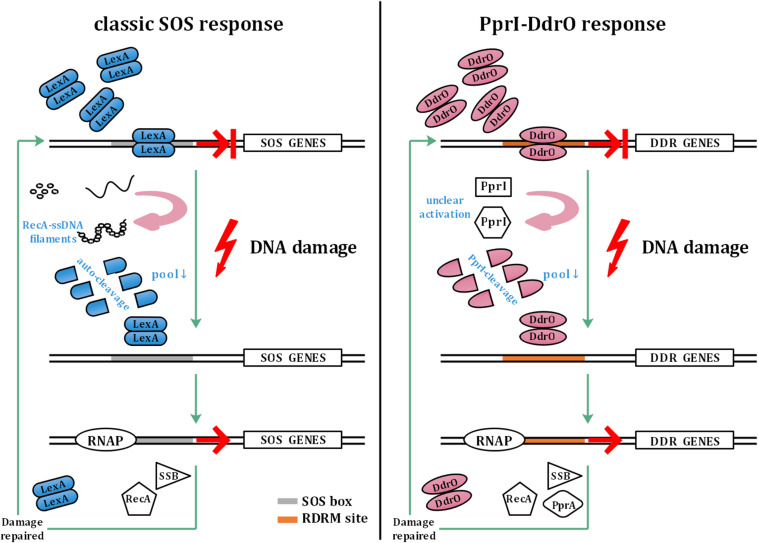
Model of the classic SOS response and PprI–DdrO response. The left panel shows the model of the classic SOS response. LexA inhibits the transcription of SOS genes under normal conditions. Sensing DNA damage, RecA forms filaments with ssDNA and activates autocleavage of LexA. With the decrease in the cellular pool of LexA, SOS box-bound LexA dissociates to initiate the transcription of SOS genes by RNAP (RNA polymerase), which are repressed again by abundant LexA after the damage is repaired. The right panel shows the model of the PprI–DdrO response for comparison. Similar to LexA, DdrO represses the transcription of DDR genes before stresses occur. PprI is activated to efficiently cleave DdrO through an unclear mechanism after sensing DNA damage signals. DDR genes such as *recA*, *ssB*, and *pprA* are upregulated until the damage is repaired.

The SOS response involves a common mechanism that is induced after DNA damage occurs in various bacteria (Radman, 1975; Butala et al., 2008, 2011). In the SOS response system, LexA functions as a transcriptional repressor that mediates the transcription of *recA* and other SOS genes. When sensing DNA damage, RecA forms filaments with ssDNA in the presence of ATP, causing the autocleavage of LexA. The decrease in the cellular pool of LexA leads to dissociation of SOS box-bound LexA, thus initiating SOS gene transcription. The upregulation of SOS genes is repressed by abundant LexA after the damage is repaired (Butala et al., 2008, 2011; Figure 1).

As one of the most radio-resistant organisms on Earth, bacteria belonging to the genus *Deinococcus* can withstand a series of environmental stresses, such as high doses of ionizing radiation, UV radiation, oxidation, mitomycin C, and long periods of desiccation, due to their extraordinary antioxidant system and DNA repair capability (Cox and Battista, 2005; Makarova et al., 2007; Blasius et al., 2008; Slade and Radman, 2011; Lim et al., 2019; Qi et al., 2020). Indeed, members of *Deinococcus* produce several antioxidants, including catalase, peroxidase, superoxide dismutase, carotenoids, and manganese ion antioxidant complex, to deal with oxidative stresses (Slade and Radman, 2011; Sharma et al., 2017). *Deinococcus* seldom invoke translesion synthesis and non-homologous end joining, but rather adopt homologous recombination to guarantee the fidelity of DNA repair (Slade and Radman, 2011). Several proteins, such as PprI, PprA, DrRRA, and OxyR, have been identified involving in the DNA damage response: PprI is the switch mediating the transcription of DDR genes, PprA contributes to UV radiation resistance and interacts with both DraTopoIB and the Gyrase A subunit, DrRRA cooperates with PprI and functions in gamma radiation resistance, and OxyR senses the presence of reactive oxygen species to regulate the antioxidant system (Chen et al., 2008; Wang et al., 2008, 2012, 2015; Bauermeister et al., 2009; Selvam et al., 2013; Kota et al., 2014).

However, unlike in most bacteria, the two encoded LexA in *Deinococcus radiodurans* do not participate in the induction of *recA*, although the autocleavage activity remains unchanged, indicating the malfunction of the classic SOS response system (Narumi et al., 2001; Sheng et al., 2004; Jolivet et al., 2006). Instead of the SOS system, a novel pathway has been found to be responsible for the DNA damage response in *Deinococcus*: two conserved proteins, DdrO—the repressor and PprI—the derepressor, comprise this unique response system (Devigne et al., 2015; Wang et al., 2015; Figure 1). To activate DDR genes participating in pathways such as DNA replication and stress response, PprI cleaves DdrO to deprive its DNA-binding ability after sensing DNA damage signals by unclear activation mechanisms (Wang et al., 2015).

This article summarizes the research progress on PprI in the last few years, mainly covering its structure and function. Potential applications and probable activation mechanisms of PprI in response to DNA damage as well as other oxidative stresses are also discussed.

## PprI Structure Reveals Three Distinct Domains

As reported before, *pprI* from *D. deserti* shares 73 and 64% sequence identity with *D. geothermalis* and *D. radiodurans* homologs and can complement the loss of radiation resistance of *pprI* deletion in *D. radiodurans* (Vujicic-Zagar et al., 2009). It is also demonstrated that PprI from either *D. geothermalis* or *D. radiodurans* can cleave DdrO from either *D. geothermalis* or *D. radiodurans*, further demonstrating that PprI cleavage of DdrO as well as the PprI–DdrO response system is conserved among *Deinococcus* species (Lu et al., 2019).

The crystal structure of PprI from *D. deserti* was solved by Vujicic-Zagar et al. (2009), revealing that the protein consists of three domains: one zinc peptidase-like domain, one helix-turn-helix motif, and one GAF-like domain. The N-terminal domain of PprI exhibits a zinc metallopeptidase fold and contains a conserved HEXXH sequence (Vujicic-Zagar et al., 2009). Ludanyi et al. (2014) and Wang et al. (2015) later proved that PprI functions as a protease targeting DdrO. Subsequent research on HEXXH-related residues has indicated that H82, E83, H86, and E113 are indispensable for metal ion binding as well as the PprI protease function (Wang et al., 2015).

The middle region of PprI comprises an HTH domain that is usually responsible for DNA binding. Although some researchers doubt the DNA-binding ability of PprI based on the structural domain arrangement and alignment with ParB-DNA structure, Lu verified the promoter-binding ability of PprI *in vitro* and *in vivo* (Vujicic-Zagar et al., 2009; Lu et al., 2012). He also proved that DNA binding is important for PprI function in response to DNA damage, and truncation of the HTH domain leads to loss of DNA affinity in PprI and the failure of RecA induction after radiation, as well as a decrease in stress resistance in *D. radiodurans* (Lu et al., 2012).

The C-terminus of PprI forms a GAF-like domain, which is one of the most widespread small molecule-binding domains responsible for binding allosteric regulatory molecules, named after a series of proteins consisting of GAF domains: cGMP-specific phosphodiesterases, adenylyl cyclases, and FhlA (Aravind and Ponting, 1997). Structural alignments revealed that the C-terminus is similar to the GAF domain in the *Thermotoga maritima* transcription factor IclR, *Klebsiella pneumoniae* CitA28, and *Escherichia coli* PDE2A that always participate in the binding of regulatory molecules such as cAMP and cGMP for the stress response. The comparison indicated that the C-terminus may be responsible for signal transduction, although this possibility needs further verification (Martinez et al., 2002; Sevvana et al., 2008).

## PprI Is a General Switch Regulating DDR Genes

Seventeen years ago, both Earl and Hua found that the *pprI* (also called *irrE*) gene can regulate the expression of *recA* gene and that its deletion would lead to the sensitivity of *D. radiodurans* to radiation (Earl et al., 2002; Hua et al., 2003; Gao et al., 2005). The disruption of *pprI* led to the decrease in resistance to gamma radiation, UV radiation, H_2_O_2_, and mitomycin C and left DdrO un-cleaved after radiation, while similar phenotypes were also detected in *D. deserti* (Vujicic-Zagar et al., 2009; Lu et al., 2012; Ludanyi et al., 2014; Wang et al., 2015).

To uncover the function role and regulation pathways of *pprI* in *Deinococcus*, proteomics and transcriptomics studies have been conducted. Proteomics research has revealed that various proteins are significantly upregulated by PprI after exposure to a low dose of gamma radiation. These proteins are involved in several different pathways, including DNA replication and repair, stress response, energy metabolism, transcriptional regulation, signal transduction, protein turnover, and chaperone functions (Lu et al., 2009). Later, microarrays and time-course sampling were applied to analyze the dynamic transcription of the *pprI* mutant strains compared with wild type. A total of 210 genes were found to be significantly induced in irradiated wild-type *D. radiodurans* but not in the irradiated *pprI* mutant strains. Consistent with the proteomics data, these genes participate in various pathways, indicating that *pprI* is a global regulator (Lu et al., 2012). Part of these genes are regulated by PprI directly, such as *pprA*, *ssB*, and *recA*, whose promoter contains RDRM (radiation/desiccation response motif) site and repressed by DdrO (Liu et al., 2003; Makarova et al., 2007). Genes like *DR0997* (*ddrI*) and *DR1114* (*hsp20*) that are also regulated by PprI without the conserved RDRM site are classified as indirectly regulated by PprI (Makarova et al., 2007; Lu et al., 2009; Singh et al., 2014; Yang et al., 2016). Moreover, Wang reported that DrRRA and PprI may collaborate to defend against environmental stresses (Wang et al., 2008, 2012).

## Repression and Derepression Mechanisms of the PprI–DdrO System

It is reported that proteins of COG2856, such as YdcM, tend to fuse with XRE (xenobiotic-response element) family proteins to form operons regulating cascade downstream. PprI is also belonging to COG2856, indicating the cooperation with an XRE family protein similar to other proteins in toxin–antitoxin systems (TAS) (Bose et al., 2008; Makarova et al., 2009). The mechanism by which PprI regulates a series of DDR genes is revealed along with the discovery of its action on the transcription repressor DdrO (Ludanyi et al., 2014; Wang et al., 2015). DdrO, a component in this DNA damage response system, belonging to the XRE family, is a transcriptional repressor that forms dimers and specifically binds to the promoter region of DDR genes, including *ddrO* itself, to repress DDR gene transcription under normal conditions (de Groot et al., 2019; Lu et al., 2019). These promoter regions contain a conserved 17-bp palindromic motif named RDRM (Makarova et al., 2007). After sensing DNA damage, DdrO is cleaved by PprI, which in turn relieves the transcriptional repression of DNA damage response genes. Thus, the repressor DdrO, in coordination with the protease PprI, constitutes the novel pathway mediating the DNA damage response in *Deinococcus*.

The detail of how the repressor DdrO works in the system remained unclear until the structure of DdrO was determined. The crystal structure of DdrO from *D. geothermalis* was solved by Lu et al. (2019). The results showed that DdrO is composed of eight α-helices, containing an HTH-containing N-terminal domain and a novel fold of C-terminal domain. Although the structure of DdrO and promoter DNA in complex is not yet available, comparison of DdrO with other XRE family protein complexes and biochemical studies have revealed a conserved binding mode and recognition/binding residues in the HTH motif. It is verified in the article that the solvent-exposed residues such as R22, R28, K30, Y42, and D45 in DG-DdrO are essential for binding affinity. As for the RDRM sequence recognition and binding, both variation of the conserved base pairs and length shortening impaired the binding of DG-DdrO. In conclusion, the extended dimeric interaction in DdrO is essential for binding to RDRM-containing sequences (Makarova et al., 2007; Lu et al., 2019; Chen et al., 2020). Besides, apart from this NTD dimerization, Arjan de Groot also revealed a CTD dimerization of DdrO that is quite different to the already known XRE family proteins such as SinR (de Groot et al., 2019).

Analysis of the novel fold in the DdrO C-terminus exhibits enrichment of hydrophobic residues forming a stable hydrophobic core. The cleavage destabilizes the C-terminal hydrophobic core and disrupts the DdrO dimer, terminating the transcriptional repression of DDR genes as the specific DNA affinity of DdrO requires its dimeric conformation (Lu et al., 2019). Arjan de Groot and colleagues solved the crystal structure of DdrO from *D. deserti* and reported similar conclusions (de Groot et al., 2019). In a manner similar to the derepression of LexA, it is found by Laurence Blanchard that cleavage by PprI decreases the intracellular pool of unbound DdrO, resulting in dissociation of RDRM-bound DdrO and leading to DDR gene transcription (Blanchard et al., 2017).

## Hypothetic Mechanisms of PprI Activation

**FIGURE 2:**
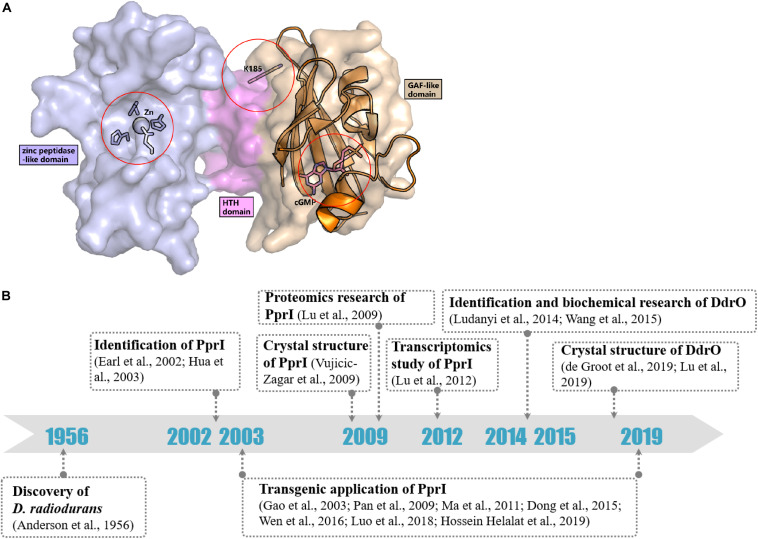
Activation hypotheses and timeline of important discoveries of PprI. **(A)** Three hypotheses of the PprI activation are present on the structure of DR_PprI, which is from the homolog modeling (Swiss model) using *D. deserti* protein (PDB: 3DTE) as the starting model. DR_PprI is shown as surface, and the zinc peptidase-like domain, helix-turn-helix motif and GAF-like domain are colored in light blue, magenta, and wheat, respectively. Related residues are shown as sticks. The aligned structure of PDE2A (RCSB PDB code 1MC0) is shown as cartoon. The four residues and metal ion in the NTD zinc peptidase-like domain represent the metal hypothesis. The Lys185 residue represents the posttranscriptional modification hypothesis. The cGMP in the GAF-like pocket by alignment represents the small molecule hypothesis. **(B)** Timeline of the important discoveries of PprI.

In contrast to most genes related to the DNA damage response, the transcriptomic study by Liu et al. (2003) detected a constant level of *pprI* transcription during the early, middle, and late phases of recovery in *D. radiodurans* after acute irradiation at 15 kGy, indicating an unclear activation mechanism of PprI. Several hypotheses have been proposed that might explain the activation of PprI since the activation mechanism of PprI after irradiation remained unknown (Figure 2A).

### The Release of Zn^2+^/Mn^2+^ Caused by Radiation or Oxidative Stress

Blanchard discovered that the protease activity of PprI from *D. deserti* could be restored in the presence of Zn^2+^, Mn^2+^, or Fe^2+^ *in vitro* (Blanchard et al., 2017). As radiation and oxidative stress can result in the rapid release of Zn^2+^ from cysteine-containing zinc sites, Qi suggested that the level of intracellular Zn^2+^ may be responsible for the activation of PprI (Maret, 2006; Kroncke and Klotz, 2009; Qi et al., 2020). Yet, it has to be demonstrated whether the level of intracellular Zn^2+^ increases. However, Wang reported that the protease activity of PprI from *D. radiodurans* depends on Mn^2+^ and that stimulation of PprI activity may rely on the alteration between Mn^2+^ and other ions (Wang et al., 2015).

### Posttranslational Modifications

Posttranslational modification (PTM) has always been thought to be responsible for activating protein function in DNA damage response pathways, such as phosphorylation of H2A.X and ubiquitylation of Ku in DNA damage signaling and the NHEJ pathway, respectively (Kinner et al., 2008; Postow et al., 2008). Recently, Zhou et al. revealed the succinylome of *D. radiodurans* that is involved in its extreme resistance. *In vitro* assays have verified that glutamate substitution of Lys185 (K185E) in PprI, which mimics lysine succinylation, results in decreased enzymatic activity but that K185A exhibits enhanced protease activity (Zhou et al., 2019; Figure 2A). Whether other kinds of modification exist in PprI and affect the activation need further research.

### Small Molecules Binding to the GAF-Like Domain

The GAF domains always bind small molecules such as cAMP and cGMP, which participate as secondary messengers in many cellular signal transduction pathways. The C-terminus of PprI exhibits a GAF-like domain, the function of which has not yet been demonstrated. Li proved that the addition of dGMP significantly enhanced *D. radiodurans* tolerance to H_2_O_2_ and gamma radiation by stimulating the activity of KatA and inducing the transcription of an extracellular nuclease (Dr_b0067) (Li et al., 2013). Based on the results, it is tempting to propose that PprI might be activated via a signaling molecule such as dGMP interacting with the GAF-like domain (Figure 2A). Regardless, the GAF domain of *D. deserti* PprI exhibits a “closed gate” (the long loop from residue 201 to residue 211 that connects the second and third strands), blocking the access to the hydrophobic pocket, which may function as a small molecule binding site (Vujicic-Zagar et al., 2009). Although this blocking may be caused by crystal stacking, unless the loop region is moved aside, otherwise, small molecules would be rejected into the pocket to activate PprI.

## Potential Applications of PprI in Genetic Engineering

It is meaningful to increase the resistance of organisms, especially industrial microbes and crops grown in extreme environments. In addition to mutant selection, overexpression of heterologous genes like the global regulator *pprI* from *Deinococcus* can help increase resistance to environmental stresses in organisms (Jin et al., 2019; Wang et al., 2019).

For example, Gao and Pan expressed *Dr_pprI* in *Escherichia coli*, which resulted in enhanced tolerances to radiation, salt, osmotic, reactive oxygen species (ROS) and other stresses. For example, the D_10_ dose of ionizing radiation increased from 50 to 250 Gy, although it is quite far from that of *Deinococcus* (Gao et al., 2003; Pan et al., 2009). Ma also found that transduction of *Dr_pprI* into ethanologenic *E. coli* increases the ethanol production by 14.7 and 26.3% from glucose and xylose, respectively (Ma et al., 2011). Similarly, Dong produced a lactic acid high-yield and stress-tolerant strain of *Lactococcus lactis* by expression of *pprI* from *D. radiodurans.* The increment of lactic acid reached even up to threefold especially under salt stress (Dong et al., 2015). Luo explored the effects of introducing *pprI* into the electrochemically active bacterium *Pseudomonas aeruginosa* PAO1 and achieved an increase in power density by 71% higher than that of the control strain (Luo et al., 2018). The transduction of *pprI* works even in eukaryotes. Hossein Helalat reported that the heterologous expression of the *pprI* gene generated a 1.5-fold alcohol and salt stress-tolerant strain of *Saccharomyces cerevisiae* (Hossein Helalat et al., 2019). Furthermore, Wen attempted to introduce *pprI* cloned in the pEGFP-c1 vector into mouse and human cells, and showed that its expression relieved acute radiation-induced damage to different organs and increased nearly 30% the survival rate by regulating expression of Rad51 (Wen et al., 2016). The detailed mechanism of how PprI affects the stress tolerance of other organisms remains unknown so far. One possible explanation is the similarity in analogous stress regulon systems. Adding PprI from *Deinococcus* may increase the amount of PprI-like protease copies and improve the survival in extreme environments to some point. The transcriptome and proteome in *E. coli* expressing PprI revealed the regulation of gene response not only to DNA damage but also to pH stress, and osmotic and oxidative stress, which also indicated the analogous stress regulon systems in *E. coli* (Zhou et al., 2011; Chen et al., 2012; Zhao et al., 2015). What is more, researchers have also inferred that PprI has other functions in addition to acting as a protease to derepress transcription in response to DNA damage. Further study will help to better reveal its functional mechanisms and can be applied to various human production activities.

## Conclusion

Identified nearly 64 years ago, *D. radiodurans* has been designated as one of the most radio-resistant organisms on Earth (Anderson et al., 1956). The reason for its robust viability has been revealed with research progresses of the antioxidant system and DNA damage repair, especially when the essential of *pprI* for the stress resistance and its orchestrating on DNA damage genes such as *recA* are confirmed (Earl et al., 2002; Hua et al., 2003; Longtin, 2003).

In the past 17 years, the *pprI* gene has been studied by genomics, transcriptomics, proteomics, bioinformatics, molecular biology, and structural biology approaches, revealing its structural and functional characteristics (Figure 2B). Structural data reveal the composition of three domains, suggesting its function as a protease, which was later demonstrated with the discovery of its specific substrate, DdrO. DNA microarrays and proteomics analysis have revealed that the *pprI* gene is responsible for regulating various genes participating in transcription, translation, metabolism, and DNA damage repair. However, transcriptomics data also suggest that the DNA damage response mediated by PprI does not rely on the induction of protein translation but on an unclear activation mechanism that needs further research.

Comparison of the PprI–DdrO response system with the SOS response system reveals distinctions between them. For one thing, the dimerization of the two repressors depends on the interaction from both NTD and CTD, while the interface of DdrO is much more extensive and CTD dependent (Lu et al., 2019; Chen et al., 2020). For another, the dissociation of LexA relies on the autocleavage, which is promoted by the stabilization of autocleavage conformation when RecA is activated after sensing DNA damage and form RecA-ssDNA-ATP filaments (Butala et al., 2008, 2011). On this occasion, the cleavage conducted by PprI is much more direct and efficient compared with the co-protease activity of RecA.

The efficiency of the PprI–DdrO response system also relies on the antioxidant intracellular environment protecting the proteome, which is provided and kept by the extraordinary antioxidant system (Daly et al., 2004, 2010; Slade and Radman, 2011). The domestication of the high-resistant *E. coli* by 100-cycle selection exhibits reduced level of hydroxylation, which further indicates the relationship between DNA damage repair and antioxidant system (Bruckbauer et al., 2020). In other words, both DDR system and antioxidant system are important, without which the radiation resistance will be greatly impaired.

DNA damage occurs throughout the entire life cycle, inducing mutation, cancer and cell death, which are prevented by the DNA damage response that includes a series of activities such as DNA repair, cell cycle checkpoints, and apoptosis. Studies on the DNA damage response can contribute to the development of new drugs for cancer therapy, such as small molecule inhibitors that target key proteins in DNA damage response and repair pathways (Li et al., 2020). Furthermore, greater knowledge of DNA damage response mechanisms may help to prevent cancer-inducing habits and guide healthy living. Research on the DNA damage response, such as the SOS and PprI–DdrO response systems, can help in elucidating the extraordinary resistance of *Deinococcus* and the mechanisms of organisms that can survive environmental stresses. Regardless, much work is needed to fully understand the multiple DNA damage response systems.

## Author Contributions

HL and YH reviewed the literature and wrote the manuscript. Both authors contributed to the article and approved the submitted version.

## Conflict of Interest

The authors declare that the research was conducted in the absence of any commercial or financial relationships that could be construed as a potential conflict of interest.
